# Kuei-Lu-Er-Xian-Jiao extract enhances BMP-2 production in osteoblasts

**DOI:** 10.1051/bmdcn/2017070102

**Published:** 2017-03-03

**Authors:** Min-Huan Wu, Ting-Hsuan Lee, Hsiang-Ping Lee, Te-Mao Li, I-Tee Lee, Po-Chuen Shieh, Chih-Hsin Tang

**Affiliations:** 1 Physical Education Office, Tunghai University Taichung 407 Taiwan; 2 Sports Recreation and Health Management Continuing Studies, Tunghai University Taichung 407 Taiwan; 3 School of Pharmacy, Tajen University Pingtung 907 Taiwan; 4 Graduate Institute of Chinese Medicine, China Medical University Taichung 404 Taiwan; 5 Department of Chinese Medicine, China Medical University Hospital Taichung 404 Taiwan; 6 Division of Endocrinology and Metabolism, Department of Internal Medicine, Taichung Veterans General Hospital Taichung 407 Taiwan; 7 Graduate Institute of Basic Medical Science, China Medical University Taichung 404 Taiwan; 8 Department of Pharmacology, School of Medicine, China Medical University Taichung 404 Taiwan; 9 Department of Biotechnology, College of Health Science, Asia University Taichung 413 Taiwan

**Keywords:** Kuei-Lu-Er-Xian-Jiao, Osteoblasts, BMP-2, ALP activity China Medical University

## Abstract

Osteoporosis is a common skeletal disorder, resulting from an imbalance in bone resorption relative to formation. Bone morphogenetic protein (BMP) is a key regulator in bone formation and osteoblastic differentiation. Hence, compounds that promote BMP expression may be suitable candidates for osteoporosis treatment. This study examined the effects of the traditional Chinese medicinal agent, Kuei-Lu-Er-Xian-Jiao (KLEXJ), on BMP-2 production in osteoblasts. We found that KLEXJ extract promoted osteoblastic differentiation marker ALP activity and increased BMP-2 production; pretreatment with PI3K and Akt inhibitors, or small interfering RNA (siRNA), reduced these effects. KLEXJ also enhanced PI3K and Akt phosphorylation. Treatment of osteoblastic cells with NF-κB inhibitors (TPCK or PDTC) markedly inhibited KLEXJ-enhancement of ALP activity and BMP-2 production. KLEXJ also significantly promoted p65 phosphorylation, while treatment with PI3K and Akt inhibitors antagonized KLEXJ-enhanced p65 phosphorylation. Thus, KLEXJ enhances ALP activity and BMP-2 production of osteoblasts through the PI3K/Akt/ NF-κB signaling pathway and hence may be suitable in the treatment of osteoporosis.

**Abbreviations**
ALP: alkaline phosphatase
BMP: bone morphogenetic protein
KLEXJ: Kuei-Lu-Er-Xian-Jiao
TCM: Traditional Chinese Medicine
FBS: fetal bovine serum
qPCR: quantitative real-time polymerase chain reaction

## 1. Introduction

Bone is a mineralized organ containing several types of cells, including osteoblasts (bone-forming cells) and osteoclasts (bone-resorbing cells), which subject bone to a continuous renewal and repair process during the life of each individual by the process of bone remodelling [1, 2]. Bone resorption and osteogenic functions must be in balance in order to maintain a constant bone mass [3, 4]. Compounds that promote osteoblastic proliferation or enhance differentiation of osteoblasts result in increased bone formation [5–7]. At this time, teriparatide^®^, the recombinant 1-34 fragment of human parathyroid hormone (rhPTH1-34), is the first bone formation agent to be approved for the treatment of osteoporosis [8, 9].

Up until now, the detailed molecular mechanism of osteoporosis has remained unclear, albeit the process is probably correlated with reduced availability or activity of growth factors. For example, bone morphogenetic proteins (BMPs), [10] were first discovered due to their ability to promote bone formation in rodents. The protein structure of BMPs resembles that of the transforming growth factor-β superfamily [11]. It is known that BMP-2 plays a critical role in osteoblastic diffemtiation and bone formation by increasing osteopontin, collagen and proteoglycan production, as well as promoting alkaline phosphatase (ALP) activity [12]. Previous research has also linked osteoporosis with specific polymorphisms in the *BMP-2* gene, confirming an association with osteoporosis [13].

Traditional Chinese Medicine (TCM) is a popular component of health care in Taiwan that provides one therapeutic option for osteoporosis treatment. Emerging studies indicate that TCM promotes bone formation and prevents bone loss in the ovariectomized rat model [14, 15]. The TCM drug Kuei-Lu-Er-Xian-Jiao (KLEXJ) is a multicomponent Chinese herbal supplement that has been used for treatment of degenerative joint diseases without adverse effects for over 2, 000 years [16, 17]. However, its role in osteoblastic function remains largely unknown. We report that KLEXJ extract increases osteoblastic differentiation marker ALP activity and BMP-2 production in osteoblasts, while simultaneously mediating the PI3 K/Akt-NF-κB pathway. Our findings suggest that KLEXJ may be useful in the treatment of osteoporosis.

## 2. Experimental section

### 2.1. Materials

Kuei-Lu-Er-Xian-Jiao (KLEXJ) contains Testudinis Plastrum (species: *Chinemys reevesii;* Animal part: plastrum); Cervi cornu (species: *Cervus elaphus;* animal part: antler); Radix Ginseng (species: *Panax ginseng* C. A. Meyer; plant part: root) and Lycii fructus (species: *Lycium barbarum;* plant part: fruit) and was prepared as follows: Testudinis Plastrum and Cervi cornu were stewed for 7 days, after which Radix Ginseng and Lycii fructus were added into the mixture. A 6.25 g extract was derived from the ratio between the 4 components, consisting of about 5 g of Testudinis Plastrum, 10 g of Cornu cervi, 0.55 g of Radix Ginseng, 1.1 g of Lycii fructus, which was provided by the LiAn Biotechnology Pharmaceutical Company (Tainan; Taiwan). Li-An Biotechnology Pharmaceutical Company was awarded the Good Manufacturing Practice certification in Taiwan (Drug license number-013857, issued by the Department of Health, Taiwan). Rabbit monoclonal antibodies specific for BMP-2, p85, Akt, p65, p-p85, p-Akt, p-p65 and b-actin, as well as anti-mouse and anti-rabbit IgG-conjugated horseradish peroxidase, were all purchased from Santa Cruz Biotechnology (Santa Cruz, CA, USA). The BMP-2 ELISA kit was obtained from Biosource Technology (Nivelles, Belgium). TRIzol reagent, Lipofectamine 2000, and the MMLV RT kit were obtained from Invitrogen (Carlsbad, CA, USA). The control, p85 and Akt siRNA were obtained from Dharmacon Research (Lafayette, CO, USA). The TaqMan assay kit was obtained from Thermo Fisher Scientific (Grand Island, NY, USA). LY294002 and other pharmacological inhibitors were purchased from Sigma-Aldrich (St. Louis, MO, USA).

### 2.2. Cell culture

The mouse osteoblast cell line MC3T3-E1 was obtained from American Type Culture Collection (Manassas, VA, USA). Cells were maintained in humidified air containing 5% CO_2_ at 37°C with a-minimum essential medium (MEM), 10% fetal bovine serum (FBS), 100 units/*ml* penicillin and 100 mg/*ml* streptomycin (Gibco-BRL Life technologies; Grand Island, NY, USA).

### 2.3. ALP activity assay

Osteoblasts were treated with KLEXJ for 24 h and then resolved with 0.2% Nonidet P-40. The medium was collected and ALP activity was examined by a commercial ALP activity detection kit (Sigma-Aldrich, St. Louis, MO, US) following manufacturer’s instructions.

### 2.4. Western blotting

Cellular lysates were prepared as our prior study [18–20]. Proteins were resolved by SDS-polyacrylamide gel electrophoresis and then transferred to polyvinyldifluoride membranes. The blot membranes were blocked with 4% non-fat milk for 1 h at room temperature, followed by incubation with primary antibodies at 4°C for overnight. After washing three times, the blots were incubated with anti-rabbit or anti-mouse HRP-conjugated secondary antibodies for 1 h at room temperature. Finally, the blots were visualized by enhanced chemiluminescence, using a Fujifilm LAS-3000 chemiluminescence detection system (Fujifilm; Tokyo, Japan).

### 2.5. Quantitative real-time polymerase chain reaction (qPCR)

Total RNA was extracted from MC3T3-E1 cells using TRIzol reagent. Messenger RNA was reversely transcribed to complementary DNA using an MMLV RT kit, and qPCR was then performed using the Taqman assay kit [21].

### 2.6. Statistical analysis

Data are presented as mean ± standard error of mean (SEM). Statistical analysis of both samples used the Student’s *t* test. Statistical comparisons of more than two groups were performed by oneway analysis of variance with Bonferroni’s post-hoc test; *p* < 0.05 was considered significant.

## 3. Results

### 3.1. KLEXJ enhances ALP activity and BMP-2 production in osteoblasts

**Fig. 1:**
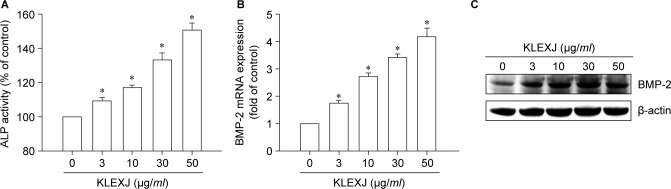
KLEXJ extract enhances ALP activity and BMP-2 expression in osteoblasts. (A) Osteoblasts were treated with KLEXJ for 48 h and ALP activity was examined by a commercial ALP assay kit. (B&C) Osteoblasts were treated with KLEXJ for 24 h and BMP-2 expression was examined by qPCR and Western blot analysis. Results are expressed as mean ± S.E.M.*, *p* < 0.05 compared with control.

Differentiated osteoblasts express high ALP activity, rendering ALP activity a key marker for osteoblastic formation [22, 23]. When we examined the role of KLEXJ in ALP activity, we found that incubation of osteoblasts with KLEXJ significantly augmented ALP activity (Fig. 1A). As BMP-2 has been reported to play a key role in osteoblastic differentiation [10], we next examined whether KLEXJ promotes osteoblastic differentiation by regulating BMP-2 expression. We found that incubation with KLEXJ stimulated BMP-2 mRNA expression and protein production, in a concentration-dependent manner (Fig. 1B&C). These combined findings indicate that KLEXJ promotes osteoblastic differentiation marker ALP activity and BMP-2 production in osteoblasts.

### 3.1. KLEXJ enhances ALP activity and BMP-2 production through the PI3K/Akt signaling pathway

**Fig. 2:**
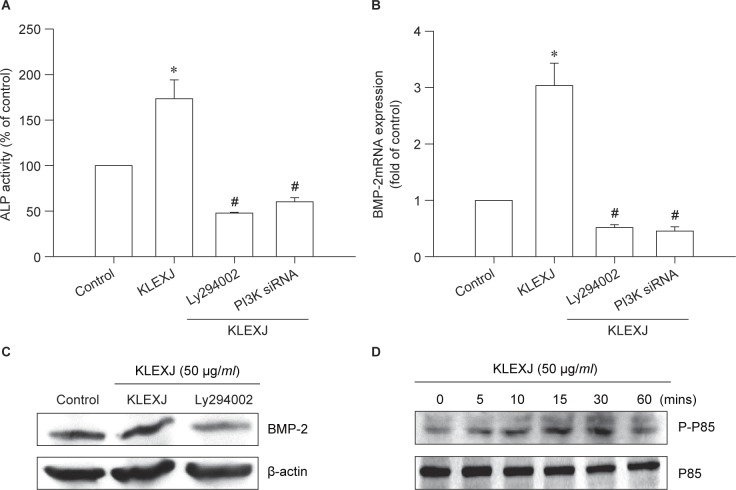
KLEXJ extract promotes ALP activity and BMP-2 expression through the PI3K pathway. (A) Osteoblasts were pretreated with Ly294002 (10 μM) for 30 min or transfected with p85 siRNA for 24 h, followed by stimulation with KLEXJ for 48 h; ALP activity was examined with a commercial ALP assay kit. (B&C) Osteoblasts were pretreated with Ly294002 (10 μM) for 30 min or transfected with p85 siRNA for 24 h, followed by stimulation with KLEXJ for 24 h; BMP-2 expression was examined by qPCR and Western blot analysis. (D) Osteoblasts were incubated with KLEXJ for indicated time intervals and p85 phosphorylation was examined by Western blot analysis. Results are expressed as mean ± S.E.M.*, *p* < 0.05 compared with control. #, *p* < 0 .05 compared with KLEXJ-treated group.

PI3K activation has been described as mediating bone formation and differentiation [24, 25]. The effects of KLEXJ were tested on the PI3K pathway. Incubation with the PI3K inhibitor Ly294002 or transfection with PI3K siRNA markedly abolished KLEXJ-induced ALP activity and BMP-2 expression (Fig. 2A-C). Treatment of osteoblasts with KLEXJ promoted phosphorylation of PI3K in a time-dependent manner (Fig. 2D). Thus, KLEXJ enhances BMP-2 production in osteoblasts *via* PI3K activation.

**Fig. 3:**
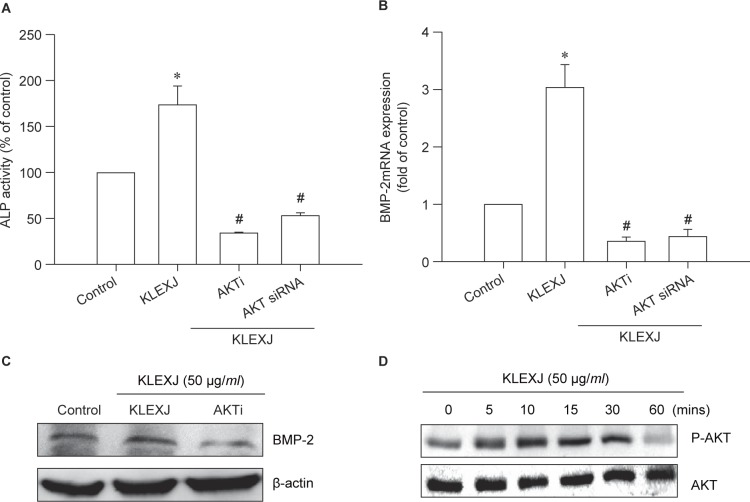
KLEXJ extract promotes ALP activity and BMP-2 expression through the Akt pathway. (A) Osteoblasts were pretreated with an Akt inhibitor (10 μM) for 30 min or transfected with Akt siRNA for 24 h, followed by stimulation with KLEXJ for 48 h. ALP activity was examined using a commercial ALP assay kit. (B&C) Osteoblasts were pretreated with an Akt inhibitor (10 μM) for 30 min or transfected with Akt siRNA for 24 h, followed by stimulation with KLEXJ for 24 h. BMP-2 expression was examined by qPCR and Western blot analysis. (D) Osteoblasts were incubated with KLEXJ for indicated time intervals and Akt phosphorylation was examined by Western blot analysis. Results are expressed as mean ± S.E.M.*, *p* < 0.05 compared with control. #, *p* < 0.05 compared with KLEXJ-treated group.

Akt is a downstream pathway in PI3K signaling and plays an important role in osteoblastic function [14, 26]. We therefore studied whether KLEXJ also activates the Akt signaling pathway. We found that an Akt inhibitor or Akt siRNA abolished KLEXJ-induced ALP activity and BMP-2 production (Fig. 3A-C). In addition, Akt phosphorylation was increased after KLEXJ stimula tion (Fig. 3D), suggesting that KLEXJ enhances ALP activity and BMP-2 production in osteoblasts through the Akt pathway.

### 3.2. KLEXJ increases ALP activity and BMP-2 production in osteoblasts *via* the NF-κB pathway

**Fig. 4:**
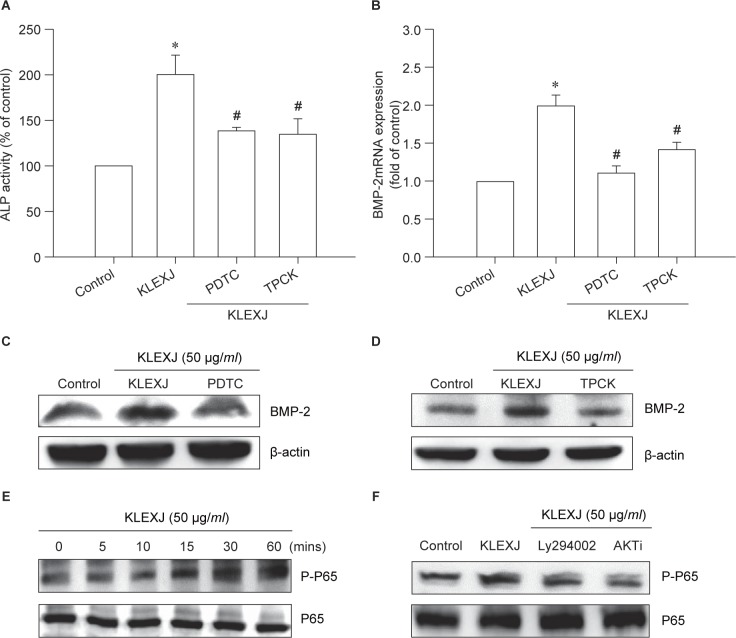
KLEXJ promotes ALP activity and BMP-2 expression through the NF-κB pathway. (A) Osteoblasts were pretreated with TPCK (10 μM) or PDTC (10 μM) for 30 min, followed by stimulation with KLEXJ for 48 h. ALP activity was examined using a commercial ALP assay kit. (B-D) Osteoblasts were pretreated with TPCK (10 μM) or PDTC (10 μM) for 30 min, followed by stimulation with KLEXJ for 24 h and BMP-2 expression was examined by qPCR and Western blot analysis. (E) Osteoblasts were incubated with KLEXJ for indicated time intervals and p65 phosphorylation was examined by Western blot analysis. (F) Osteoblasts were pretreated with Ly294002 or an Akt inhibitor, followed by stimulation with KLEXJ. p65 phosphorylation was examined by Western blot analysis. Results are expressed as mean ± S.E.M. *, *P* < 0. 05 compared with control. #, *p* < 0.05 compared with KLEXJ-treated group.

NF-κB activation has been indicated to regulate BMP-2 expression and is implicated in bone formation [27, 28]. We assessed whether NF-κB activation is mediated *via* KLEXJ increasing BMP-2 production. Pretreatment with NF-κB inhibitors (TPCK and PDTC) reversed KLEXJ-induced ALP activity and increases in BMP-2 expression (Fig. 4A-D). Furthermore, KLEXJ increased phosphorylation of p65 in a time-dependent manner (Fig. 4E). In contrast, KLEXJ-induced activation of p65 was antagonized by pretreatment with Ly294002 and an Akt inhibitor (Fig. 4F). Hence, NF-κB is the downstream molecule of PI3K/Akt.

## 4. Discussion

Kuei-Lu-Er-Xian-Jiao (KLEXJ), a TCM formula, is widely used in traditional medicine for osteoporosis treatment and has been reported to reduce osteoarthritis progress [16]. However, the detailed effects of KLEXJ in bone cells are unclear. To the best of our knowledge, this study is the first analysis of the role played by KLEXJ extract in osteoblasts. Our results demonstrate that

KLEXJ extract induces ALP activity (an important osteoblastic differentiation marker). In addition, we suggest that BMP-2 acts as a target molecule of KLEXJ-induced signaling that requires the PI3K/Akt/NF-κB pathway.

The detailed molecular mechanisms of osteoporosis remain unknown, but they are believed to correlate with decreased availability or activity of bone growth factors: *e.g.,* BMPs that play key roles in bone remodeling and formation [29, 30]. The present study found that KLEXJ extract increases production of BMP-2. In contrast, incubation of osteoblasts with KLEXJ increased osteoblastic differentiation marker expression (ALP activity). These results imply that KLEXJ promotes bone formation by up-regulating expression of BMP-2.

PI3K activation is a potential signaling pathway that regulates bone formation [31, 32]. Here, we report that both a PI3K inhibitor and a siRNA antagonized KLEXJ-promoted activity of ALP and production of BMP-2. Incubation of osteoblasts with KLEXJ increased phosphorylation of PI3K, suggesting that PI3K activation plays a crucial role in KLEXJ-increased bone differentiation and BMP-2 expression.

Akt activation is reportedly mediates ALP activity during osteoblastic cell differentiation [33, 34]. In this study, we found that KLEXJ promotes phosphorylation of Akt, while an Akt inhibitor or siRNA diminishes KLEXJ-induced potentiation of ALP activity and BMP-2 production in osteoblasts, which suggests that Akt activation plays a critical role in KLEXJ-promoted osteoblastic function.

The BMP-2 5’ promoter region contains the ERE, AP-1 and Sp1 binding sites, which regulate BMP-2 expression [35]. In this study, we found that the NF-κB inhibitors PDTC and TPCK antagonized KLEXJ-induced increases in ALP activity and BMP-2 expression in osteoblasts. In addition, treatment with KLEXJ enhanced p65 NF-κB phosphorylation, suggesting that NF-κB activation plays a critical role in KLEXJ-promoted bone formation and BMP-2 production. Further evidence that activation of NF-κB plays a key role in TCM-mediated BMP-2 production is shown by the TCM formula Si-Wu-Tang, which also increases BMP-2 expression through NF-kB activation [14]; moreover, the Chinese herbal medicine *Cistanche deserticola* extract up-regulates BMP-2, which involves the NF-kB pathway [23]. In this study, we found that PI3K and Akt inhibitors diminished KLEXJ-induced increases in p65 phosphorylation. Therefore, the PI3K/ Akt pathway is mediated by KLEXJ-promoted NF-κB activation.

## 5. Conclusions

Drugs that are synthesised from natural products have a key role in pharmaceutical care [36–38] and have proven to be critical sources of potential products for osteoporosis treatment [39, 40]. We have shown that KLEXJ boosts ALP activity and BMP-2 production in osteoblasts *via* the PI3K/Akt-NF-κB pathway and hence may be suitable in the treatment of osteoporosis.

## Conflict of interest

None of the authors have any financial or personal relationships with other people or organizations that could inappropriately influence this work.
